# Extrapulmonary Sarcoidosis: A Diagnostic Challenge

**DOI:** 10.7759/cureus.11076

**Published:** 2020-10-21

**Authors:** Iftikhar Nadeem, Usman Feroze Khatana, Masood Ur Rasool, Ali Qamar, Mohammed Azher

**Affiliations:** 1 Respiratory Medicine, Lister Hospital, Stevenage, GBR; 2 Internal Medicine, Bedford Hospital, NHS Trust, Bedford, GBR; 3 Respiratory Medicine, Norfolk and Norwich University Hospital, Norwich, GBR; 4 Internal Medicine, Kettering Hospital, NHS Trust, Kettering, GBR; 5 Pulmonary Medicine, Bedford Hospital, NHS Trust, Bedford, GBR

**Keywords:** non-caseating granuloma, extrapulmonary sarcoidosis, stress fractures, ground-glass opacity

## Abstract

Extrapulmonary sarcoidosis accounts for only two percent of the total sarcoid cases. Sarcoidosis affecting the musculoskeletal system alone is even rarer. Diagnosis is based on suggestive history, clinical features, imaging followed by confirmation of non-caseating granulomas on a biopsy. Steroids form the first line of treatment for patients where musculoskeletal system is involved. We report the case of a 26-year-old gentleman who presented with right foot pain and unintentional weight loss. A magnetic resonance imaging (MRI) scan of feet confirmed bilateral stress fractures of both feet. After a battery of investigations and resultant myriad constellation of investigation findings, the diagnosis of sarcoidosis was confirmed on a tissue biopsy obtained via bronchoscopy. This case report discusses the complex journey from presentation to diagnosis and subsequent treatment while also exploring important differentials that need to be ruled out in such scenarios.

## Introduction

Sarcoidosis is a chronic inflammatory and infiltrative disorder of unknown aetiology, which can affect multiple organ systems. The typical presentation of sarcoidosis includes nonspecific symptoms such as cough, weight loss, arthralgia and shortness of breath. The true incidence of sarcoidosis is difficult to ascertain due to variation in different populations. In the United Kingdom the reported prevalence is 8% [1].

The pulmonary involvement of sarcoidosis is present in 90% of patients [2]. Eyes, skin, liver, spleen, heart, nervous system, bone and muscles can also be involved. Extra-pulmonary disease is usually associated with increased morbidity [2,3].

Extra-pulmonary sarcoidosis affecting the musculoskeletal system is extremely rare and hence can potentially be very difficult to diagnose. Steroids are the mainstay of treatment followed by immunosuppressants [1].

## Case presentation

We present the case of a 26-year-old Asian gentleman who presented to the Accident & Emergency (AnE) with three-week history of pain in the right foot. The pain was present all the time and there were no aggravating or relieving factors. He had no previous medical problems. There was no history of tobacco or alcohol use, and he was a car mechanic by profession. There was no history of trauma. He had no allergies and did not have any pets. There was no history of recent travel. Physical examination, vitals and an X-ray of right foot were normal and the patient was discharged home on simple analgesia. No blood tests were done on his first presentation.

A referral to physiotherapy was made as well. He, however, represented a week later with similar symptoms. His D-dimers were elevated, however, right leg venous Doppler was negative for a deep vein thrombosis (DVT).

He presented to AnE for the third time and in addition to the foot pain, he now reported tingling sensations in his hands and weight loss of 1 stone in last three weeks. His general practitioner (GP) was suspecting a rheumatological problem and had already requested a computed tomography of chest/abdomen/pelvis (CT CAP) and a magnetic resonance imaging (MRI) of the right foot for him.

He was admitted to the hospital on this occasion. On examination his chest was clear with a central trachea and normal vesicular breathing. There was no clubbing and there were no palpable lymph nodes. Heart sounds were normal. Abdomen was soft, non-tender with a palpable liver edge 2 cm below the costal margin. Neurological exam revealed good power and tone in all limbs with no focal motor or sensory deficit. There were no cerebellar signs and he had a normal gait.

His blood eosinophils were raised at 1.1 with deranged liver function tests (LFTs). A chest X-ray on admission was normal.

He was referred to the Neurology team for his symptoms of pain and paraesthesias. A diagnosis of multiple sclerosis was ruled out by a normal MRI head and spine. A lumbar puncture (LP) was performed and cerebrospinal fluid (CSF) showed raised protein (1.24), normal opening pressure and no oligo-clonal bands with normal differential counts. Neurology team further advised nerve conduction studies (NCS) which showed “subacute demyelinating length dependent sensorimotor polyneuropathy with substantial axonal loss in the distal lower limbs”. Neurology suspected a diagnosis of chronic inflammatory demyelinating polyneuropathy (CIDP), however, duration of his symptoms was short and inconsistent with this diagnosis. He received five days of IV immunoglobulins with little improvement. Neurology advice was to consider a course of methylprednisolone. He was booked in for outpatient neurology follow-up.

**Figure 1:**
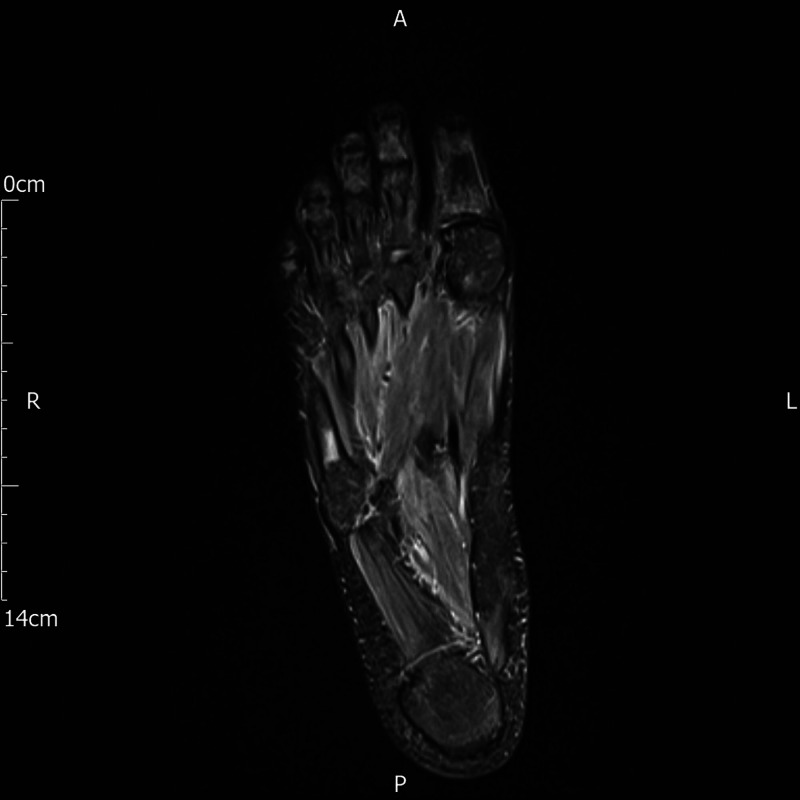
MRI right foot showing stress fracture in fifth metatarsal.

**Figure 2:**
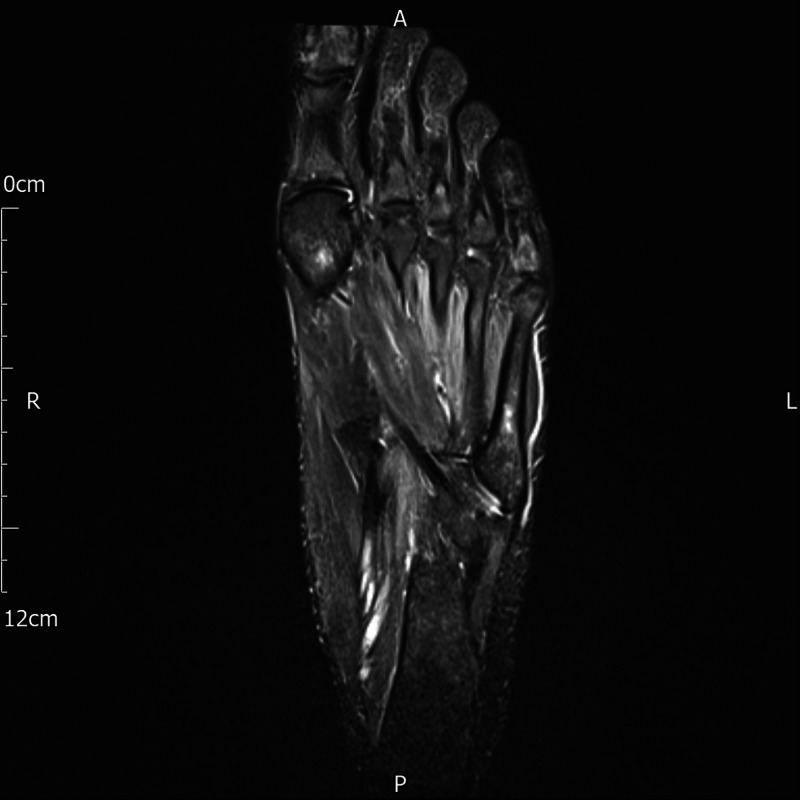
MRI left foot showing stress fractures in first, second, fourth and fifth metatarsals.

MRI (Figure 1) of his right foot showed stress fracture of fifth metatarsal and focal oedema in anterior aspect of calcaneum. Orthopedic team was called upon. They asked for an MRI of left foot which showed multiple stress-related focal changes/stress fractures in first, second, fourth and fifth metatarsals (Figure 2). They advised “off loader boots” on both feet and mobilization as tolerated. Outpatient follow-up was arranged.

Rheumatology team reviewed him and advised that he most likely has marble bone disease. They advised outpatient rheumatology follow-up post discharge.

He was referred to hematology in view of young age, weight loss and an enlarged liver. After reviewing the case hematology team conducted a bone marrow biopsy which was normal.

His liver was imaged with an ultrasound in view of deranged LFTs and palpable liver and was noted to be “enlarged in size and hypoechoic”. An acute liver screen to look for viral, autoimmune, haemochromatosis and fatty liver disease was carried out and was negative.

**Figure 3:**
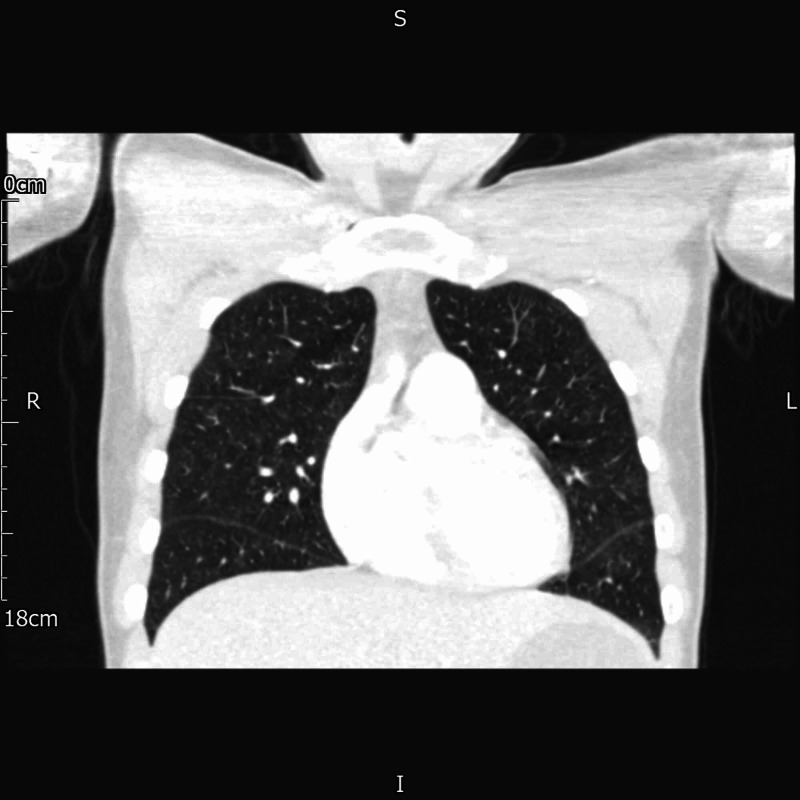
Coronal images of chest CT showing bilateral ground glass opacification.

The CT-CAP (Figure 3) done for unexplained weight loss was reported as having “multilobar bilateral patchy subtle ground glass pulmonary opacification (GGO)”.

This prompted the involvement of the respiratory team. The respiratory team advised lung function testing (PFTs) (Table 1) that showed a restrictive defect with reduced TLCO.

**Table 1:** Lung function test VC: Vital capacity; FVC: Forced vital capacity; FEV1: Force expiratory volume in one sec; PEF: Peak expiratory flow; TGV: Thoracic gas volumes; RV: Residual volume; TLC: Total lung capacity; TLCO: Transfer capacity of lung for carbon monoxide uptake; KCO: Diffusion coefficient corrected for alveolar volume.

	Predicted Values	Measured Value	Percentage Predicted	Standardised Residual
Spirometry				
VC	4.19			
FVC	4.01	3.38	84	-1.03
FEV1	3.43	3.15	92	-0.054
FEV1/FVC	86.70	93.18	107	0.90
PEF	9.10	9.06	100	-0.03
Lung Volumes				
TGV	3.03	2.65	87	-0.64
RV	1.55	1.37	88	-0.044
TLC	6.14	4.52	74	-2.31
Diffusion Test				
TLCO	10.50	6.96	66	-2.51
KCO	1.54	1.64	106	—
TLCO cor	10.50	6.96	66	-2.51
KCO cor	1.54	1.64	106	—

**Figure 4:**
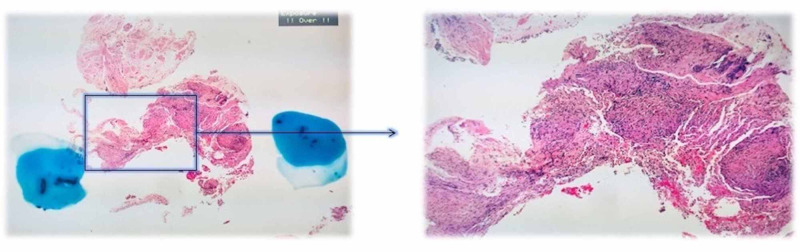
Transbronchial lung biopsy showing non-necrotising granuloma.

T-Spot was negative. In view of the eosinophilia, GGO changes on CT and lung function test findings, a bronchoscopy with transbronchial lung biopsy (TBBx) was done and bronchoalveolar lavage (BAL) samples were taken for microbiology, cytology and histopathology. A differential count revealed mononuclear cells 60%, lymphocytes 35%, neutrophils 4%, and eosinophils 1%. Aura-mine, fungal and viral cultures were negative. Trans-bronchial lung biopsy showed “non-necrotising granulomatous inflammation” (Figure 4).

A number of differentials were considered before finally treating the patient as sarcoidosis. His bloods since his first presentation through to his follow-up are tabulated below (Tables 2, 3) along with a list of other investigations and how they were used to rule out the considered differentials.

**Table 2:** Investigations WCC: White cell count; Hb: Haemoglobin; Plt: Platelets; ALP: Alkaline phosphatase; ALT: Alanine aminotransferase; GGT: Gamma-glutamyltransferase; Alb: Albumin; ACE: Angiotensin converting enzyme; AFB: Acid fast bacilli; BAL: Bronchoalveolar lavage; CSF: Cerebrospinal fluid.

Investigation	13/07/19		07/8/19	23/8/19		30/8/19		15/10/19	
WCC	11.4		9.9	9.7		8.2		8.5	
HB	127		146	134		122		148	
Plt	312		338	291		183		299	
Eosinophils	0.1		0.2	1.1		1.3		0.0	
ALP	230		229	170		-		90	
ALT	65		92	41		-		55	
Bilirubin	5		7	6		-		4	
GGT	-		-	100		-		-	
Alb	35			37		-		46	
Urea, creatinine and electrolytes & calcium	Normal			Normal		Normal		Normal	
D-dimer	2.05			-		-		-	
Other Investigations									
ACE levels					Normal				
Vasculitis screen					Negative				
HIV, Hepatitis serology					Negative				
AFB and T-spot					Negative				
Coeliac screen					Negative				
BAL					Organism not seen		Culture no growth		
CSF					Total Protein 1.24		Oligoclonal Bands not seen		
Nerve conduction studies	Subacute demyelinating length dependent sensorimotor polyneuropathy with substantial axonal loss in the distal lower limbs								

**Table 3:** List of differentials HP: Hypersensitivity pneumonitis; ANCA: Antineutrophilic cytoplasmic antibodies; PCP: Pneumocystis carinii pneumonia; BAL: Bronchoalveolar lavage; AFB: Acid fast bacilli; TBBx: Transbronchial lung biopsy.

Differentials	Ruled out by
Histoplasmosis	No history of travel to US, no lymph nodes on CT scan
Fungal infection	No fungal growth on biopsy and BAL, Aspergillus serology negative
TB	No Caseation, No AFB on BAL, T-spot negative
HP	No exposure history, Extrapulmonary involvement, No features of fibrosis on TBBx, Avian antibodies negative
Vasculitis	Negative screen, normal kidney function, no evidence of vasculitis on biopsy
Eosinophilic Granulomatosis with Polyangiitis (EGPA)	Negative ANCA, no rash, no history of asthma, no vasculitis on biopsy
Atypical pneumonia	Negative atypical pneumonia screen
Allergic bronchopulmonary aspergillosis	Normal aspergillus specific IgE, normal aspergillus precipitins
Infectious causes	Negative cultures (blood and BAL)
Pneumocystis jerovici pneumonia	Negative serology for PCP, Strongyloides, Wuchereria and Schistosoma Negative HIV and Hepatitis serology

The biopsy results in conjunction with eosinophilia, high urinary calcium, restrictive lung disease on PFTs and high proteins on LP were consistent with a diagnosis of sarcoidosis.

He was started on oral prednisolone 50 mg by the respiratory team and discharged home with a planned multidisciplinary follow-up to ensure comprehensive care.

**Figure 5:**
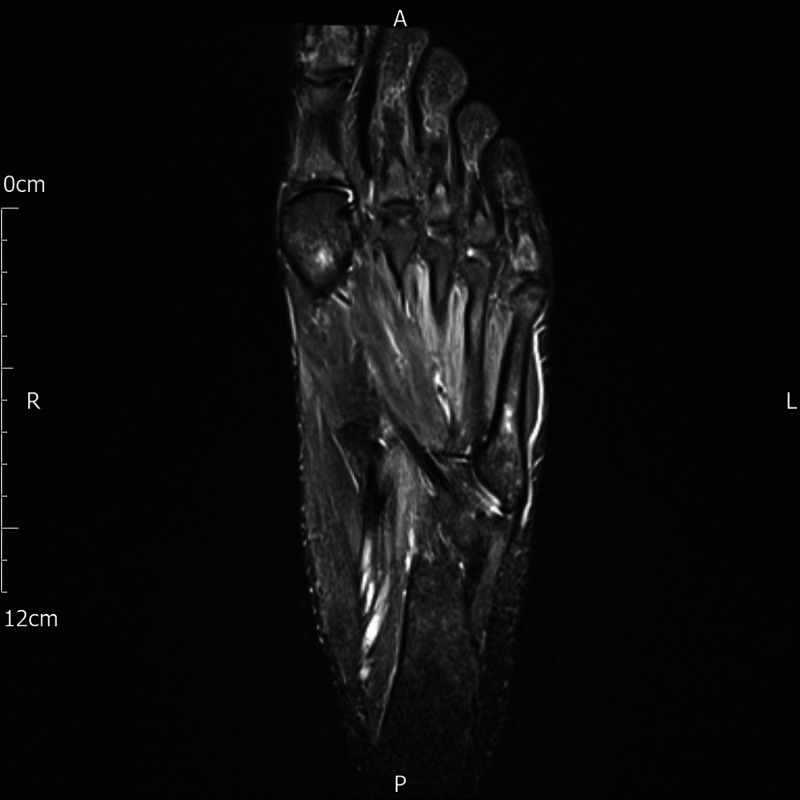
MRI left foot showing resolution in stress fractures in first, second, fourth and fifth metatarsals.

**Figure 6:**
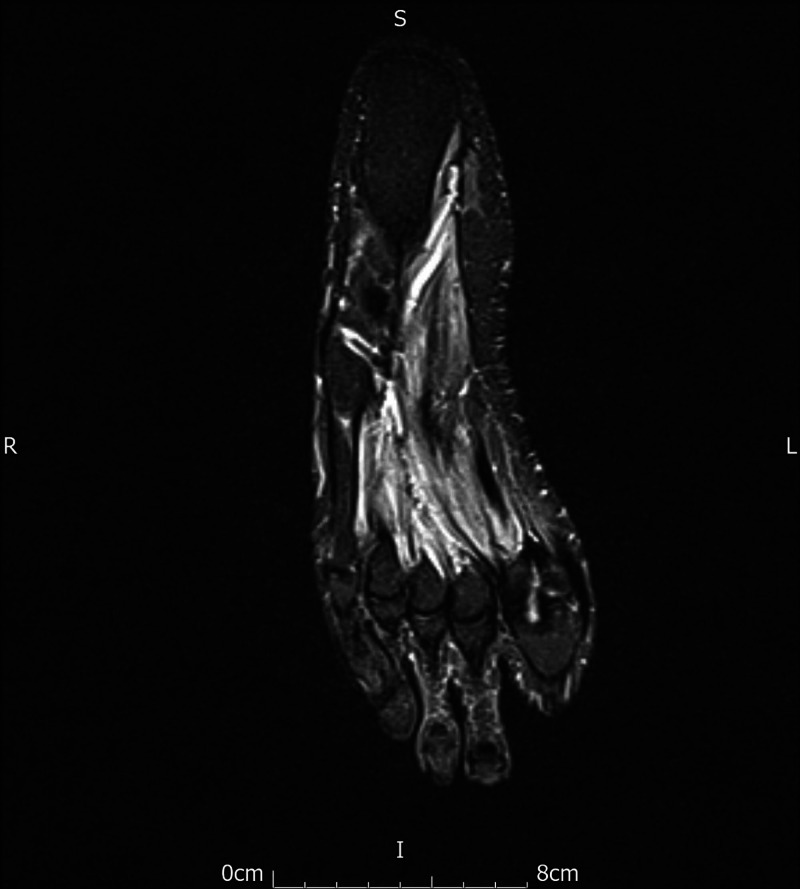
MRI right foot showing resolution of stress-related marrow oedema.

He has since been reviewed in the respiratory, rheumatology, neurology and orthopedic clinics. He reports his symptoms have shown significant improvement. His repeat MRI of feet (Figures 5, 6) two months after his discharge showed that his stress fractures had improved. He also had a repeat CT thorax, three months after discharge which showed resolution of GGO. He remains under respiratory follow-up and continues to do well.

## Discussion

Sarcoidosis is a systemic disorder resulting in granuloma development in multiple organs of the body. Pulmonary involvement occurs in 90% of the patients followed by cutaneous, ophthalmic and cardiac involvement [2]. Extra-pulmonary manifestations occur concurrently with pulmonary disease in about 50% of the cases whereas isolated extra pulmonary sarcoidosis is seen only in 2% of the patients [1]. Bone involvement in sarcoidosis is rare and is mostly associated with multisystem disease involving lung, liver, spleen and kidneys. Diagnosis of sarcoidosis requires relevant history and clinical findings plus demonstration of non-caseating granulomas. This is compounded in case of isolated symptoms in the absence of pulmonary findings. Imaging and biopsy of the affected bones are used for establishing the diagnosis. Mainstay of the treatment is glucocorticoid therapy however steroid sparing agents have also been reported in literature though they lack a strong evidence base.

Diagnosing sarcoidosis generally requires a patient with symptoms consistent with the disease and presence of granulomas demonstrated on a biopsy. This in majority of the cases is straight forward as most patients present with respiratory symptoms or signs which in combination with extra pulmonary features will guide the clinician towards a diagnosis of sarcoidosis. The diagnosis would require imaging (which normally includes chest radiographs), ACE levels and trans-bronchial biopsies. Epithelioid granulomas on trans-bronchial biopsy are the definitive diagnostic investigation.

This however becomes complicated in cases, such as the one we presented, where there is absence of typical symptoms such as cough, shortness of breath, erythema nodosum or weight loss. The diagnostic tools used are different for each system but again demonstration of the granulomas on biopsy remains the gold standard.

In our patient the presentation was longstanding foot pain. This later progressed into a vague and seemingly unrelated collection of symptoms and after multiple visits to his GP and AnE he ended up needing admission. The trouble in his case was not putting the puzzle together but actually finding all the pieces of the puzzle first. He went through extensive investigations to rule out other causes of his symptoms. One month from his initial presentation for seemingly isolated foot pain, this gentleman had stress fractures in his feet, polyneuropathy, an enlarged liver, CT evidence of ground glass changes and worsening eosinophilia on his bloods. The journey to reach a unifying diagnosis meant that a long list of differentials needed to be ruled out in addition to having a confirmation of the diagnosis on biopsy.

Bilateral stress fractures in the feet, as a presentation of sarcoid, are very rare and hence it is important to know how sarcoid involves the musculoskeletal system. The involvement of joints, bone and muscles cause a myriad of symptoms which can be categorized as acute or chronic.

Acute syndrome includes Lofgren syndrome. Lofgren is characterized by arthritis, erythema nodosum and bilateral hilar lymphadenopathy [3, 4]. This involves the ankles mostly followed by other large and small joints. Lofgren does not cause bone destruction and carries a good prognosis.

On the other hand the chronic arthritis seen in extrapulmonary sarcoidosis usually displays a symmetric, medium to large oligoarthritis [2]. Bone involvement occurs in 3 to 13% of sarcoid patients. Axial skeleton, hands and feet are most commonly affected [2, 4]. Half of the patients remain asymptomatic. Imaging shows three distinct types of changes caused by Sarcoidosis: lytic, permeative and destructive. These result in bone resorption and cyst formation, tunneled reticular appearance of cortex and severe bone damage and fractures respectively [5].

The diagnosis of musculoskeletal sarcoidosis depends upon radiological imaging. However, histopathological diagnosis is more specific and considered the gold standard [1]. MRIs have shown to be more sensitive in detecting destructive bony lesions. Diagnosis is confirmed by presence of non-caseating granulomas either in bone or other organs. Chest imaging should be considered, even in absence of respiratory symptoms as it may identify covert changes and can guide the clinician towards the diagnosis of sarcoidosis [6].

Management of acute musculoskeletal sarcoidosis involves use of nonsteroidal anti-inflammatory drugs (NSAIDs) with glucocorticoid use in up to 2/3rds of the patients. Normally, steroids should continue for three to six months with slow tapering.

On the other hand, chronic musculoskeletal disease is treated with glucocorticoids (GCs). Methotrexate, hydroxychloroquine, azathioprine, rituximab, infliximab and adalimumab have been reported in literature but due to lack of randomized control trials the evidence base is not that strong [5, 6].

## Conclusions

Bone involvement with other systemic symptoms and signs can indicate sarcoidosis even in young age. Investigations should be arranged to look at the liver, spleen, kidneys if sarcoidosis is suspected.

In such cases treatment is usually with GCs or steroid sparing agents if there is poor response to GCs.
